# PDLIM7 Synergizes With PDLIM2 and p62/Sqstm1 to Inhibit Inflammatory Signaling by Promoting Degradation of the p65 Subunit of NF-κB

**DOI:** 10.3389/fimmu.2020.01559

**Published:** 2020-08-04

**Authors:** Aya Jodo, Azusa Shibazaki, Asuka Onuma, Tsuneyasu Kaisho, Takashi Tanaka

**Affiliations:** ^1^Laboratory for Inflammatory Regulation, RIKEN Center for Integrative Medical Sciences (IMS), Yokohama, Japan; ^2^Department of Immunology, Institute of Advanced Medicine, Wakayama Medical University, Wakayama, Japan

**Keywords:** LIM protein, NF-κB, ubiquitin E3 ligase, inflammation, p62/Sqstm1

## Abstract

Activation of NF-κB transcription factors is critical for innate immune cells to induce inflammation and fight against microbial pathogens. On the other hand, the excessive and prolonged activation of NF-κB causes massive inflammatory damage to the host, suggesting that regulatory mechanisms to promptly terminate NF-κB activation are important to prevent immunopathology. We have previously reported that PDLIM2, a PDZ-LIM domain-containing protein, is a nuclear ubiquitin E3 ligase that targets the p65 subunit of NF-κB for degradation, thereby suppressing NF-κB activation. Here we show that PDLIM7, another member of LIM protein family, is also a ubiquitin E3 ligase that inhibits NF-κB-mediated inflammatory responses. PDLIM7 directly polyubiquitinates p65 and promotes its proteasomal degradation. Moreover, PDLIM7 heterodimerizes with PDLIM2 to promote synergistic PDLIM2-mediated degradation of p65. Mechanistically, PDLIM7 promotes K63-linked ubiquitination of PDLIM2 and then the proteasome/autophagosome cargo protein p62/Sqstm1 binds to both polyubiquitinated PDLIM2 and the proteasome, thereby facilitating the delivery of the NF-κB-PDLIM2 complex to the proteasome and subsequent p65 degradation. Consistently, double knockdown of PDLIM7 and either PDLIM2 or p62/Sqstm1 results in augmented proinflammatory cytokine production compared to control cells or single knockdown cells. These data delineate a new role for PDLIM7 and p62/Sqstm1 in the regulation of NF-κB signaling by bridging a ubiquitin E3 ligase and the proteasome.

## Introduction

Innate immune cells, such as macrophages and dendritic cells, recognize invading microbial pathogens by pattern recognition molecules, such as Toll-like receptors (TLRs). The transcription factor nuclear factor **κ**B (NF-**κ**B) is critically involved in TLR-mediated activation of these innate cells. In a resting state, the NF-**κ**B p65/p50 heterodimer is sequestrated in the cytoplasm by interacting with I**κ**Bα, an inhibitor of NF-**κ**B. Signaling through TLR causes the proteasomal degradation of I**κ**Bα. Heterodimers of p65 and p50 then translocate to the nucleus, bind to NF-**κ**B sites in the enhancers of multiple inflammation-related genes, including those encoding proinflammatory cytokines, and induce their transcription (1, 2). However, inflammatory responses must eventually be terminated, otherwise, excessive and prolonged activation of NF-**κ**B signaling will cause massive damage to the host and could lead to autoimmune diseases such as rheumatoid arthritis, systemic lupus erythematosus, type 1 diabetes, multiple sclerosis, and inflammatory bowel disease (3, 4). Therefore, a system to negatively regulate inflammatory signaling is crucial for preventing immunopathology (5).

PDLIM2 (also known as SLIM or mystique) is a nuclear protein containing both PDZ (postsynaptic density 65-discs large-zonula occludens 1) and LIM (abnormal cell lineage 11-isket 1-mechanosensory abnormal 3) domains (6–8). We have recently demonstrated that PDLIM2 negatively regulates NF-**κ**B activity as a nuclear ubiquitin E3 ligase targeting the p65 subunit of NF-**κ**B (9). PDLIM2 binds to p65 and facilitates its polyubiquitination via its LIM domain. In addition, PDLIM2 transports p65 to discrete intranuclear compartments, called promyelocytic leukemia protein (PML) nuclear bodies, where the polyubiquitinated p65 is degraded by the nuclear proteasome. Consistent with these findings, PDLIM2 deficiency revealed increased nuclear p65 protein, impaired p65 ubiquitination and enhanced production of proinflammatory cytokines in response to TLR stimulation. PDLIM2 belongs to a large family of LIM proteins, to date numbering more than 30 family members (10). These proteins are classified into subgroups depending on the domain structure. Seven proteins with both PDZ and LIM domains, PDLIM1/Elfin, PDLIM2, PDLIM3/ALP, PDLIM4/Ril, PDLIM5/ENH, PDLIM6/ZASP/Cipher, PDLIM7/Enigma/LMP1 comprise a PDZ-LIM protein subfamily (11, 12). The long-term goal of our laboratory is to clarify the mechanisms how these PDZ-LIM proteins regulate inflammatory responses. In this study, we demonstrate that PDLIM7 is also a ubiquitin E3 ligase that negatively regulates NF-**κ**B signaling. Notably, PDLIM7 not only bound to p65 and directly mediated its polyubiquitination and degradation, but also formed a heterodimer with PDLIM2, promoted K63-linked polyubiquitination of PDLIM2, and facilitated PDLIM2-mediated p65 degradation cooperatively with p62/Sqstm1. The p62/Sqstm1 bound to both polyubiquitinated PDLIM2 via its ubiquitin associated (UBA) domain and to the proteasome via its Phox-BEM1 (PB1) domain and promoted the transfer of the NF-**κ**B-PDLIM2 complex to the proteasome, thereby facilitating p65 degradation. Consistently, cells lacking PDLIM7, and either PDLIM2 or p62/Sqstm1, had enhanced production of proinflammatory cytokines. This study demonstrates that PDLIM7 and PDLIM2 are heterodimeric E3 ligases that synergistically inhibit NF-**κ**B signaling, whereby PDLIM7 enhances the activity of PDLIM2 to degrade p65 by the proteasome.

## Materials and Methods

### Expression Vectors

For the His-, c-Myc- and FLAG-tagged PDLIM7, mouse *Pdlim7* (GeneBank accession: NM_001114088) was subcloned into pcDNA3-His (Invitrogen), pCMV-Myc and pCMV-DYKDDDDK (Clontech), respectively. For the c-Myc-tagged ΔPDZ PDLIM7 mutant, the coding region corresponding to amino acids 80–457 of *Pdlim7* was subcloned into pCMV-Myc or pCMV-DYKDDDDK. For the ΔLIM3 PDLIM7 mutants, we mutated cysteine 400 of c-Myc- and FLAG-tagged PDLIM7 to a STOP codon, thereby only the coding region corresponding to amino acids 2–399 of *Pdlim7* is expressed. For the ΔLIM2/3 PDLIM7 mutants, we mutated cysteine 340 of c-Myc-tagged PDLIM7 to a STOP codon, thereby only the coding region corresponding to amino acids 2–339 of *Pdlim7* is expressed. His-, and c-Myc-tagged PDLIM2, c-Myc-tagged ΔPDZ, ΔLIM mutant of PDLIM2 and FLAG-tagged p65 were previously described (7, 9). For the Flag-tagged PDLIM2, mouse *Pdlim2* was subcloned into pCMV-3Tag-1 (Agilent Technologies). The FLAG- and c-Myc-tagged PDLIM2 K0 mutants were generated by mutating all nine lysines at K31, K37, K68, K149, K221, K279, K285, K318, and K331 to arginine. K48R and K63R mutants of His-tagged ubiquitin (GeneBank accession: NM_019639) were generated by mutating lysine at K48 or K63 to arginine, respectively. For K48 and K63 mutants of His-tagged ubiquitin, every lysine except K48 or K63 was changed to arginine, respectively. C-Myc-tagged PDLIM1 was previously described (13). For c-Myc-tagged PDLIM3, PDLIM4, PDLIM5, and PDLIM6, coding regions of mouse *Pdlim3* (GeneBank accession: NM_001374652), *Pdlim4* (GeneBank accession: NM_019417), *Pdlim5* (GeneBank accession: NM_019808), and *Pdlim6* (GeneBank accession: NM_011918) were subcloned into pCMV-Myc, respectively. HA- and His-tagged p62/Sqstm1 were generated by subcloning the coding sequence of mouse *Sqstm1* (GeneBank accession: NM_011018) into pCMV-HA-N (Clontech) or pcDNA3-His, respectively. For the His-tagged ΔUBA p62/Sqstm1 mutant, the coding region corresponding to amino acids 2-369 of *Sqstm1* was subcloned into pcDNA3-His. FLAG-tagged p50 expression plasmid (p50 cFLAG pcDNA3) was purchased from Addgene (#20018). The ELAM-1 luciferase reporter construct was kindly provided by D. Golenbock (14). The pRL-Null renilla construct (#E2271) was from Promega. The pGL4.32-Null renilla construct was generated by deleting promoter region from the pGL4.32-NF-**κ**B-RE renilla constructs (Promega, #E8491).

### Reagents and Antibodies

LPS (from *Salmonella enterica*; L-2262) was from Sigma. MG132 and bafilomycin A1 were purchased from Calbiochem. Murine GM-CSF was from R&D Systems. Human ligand for the receptor tyrosine kinase Flt3 (Flt3L) was purchased from Peprotech. Anti-DYKDDDDK (NU01102) antibody was from Nacalai USA and used as anti-FLAG antibody. Anti-p65 (sc-372), p50 (sc-7178), I**κ**Bα (sc-371), HSP90 (sc-7947), Lamin B (sc-6217) antibodies and Omni-probe (sc-7270; used as anti-His) were purchased from Santa Cruz Biotechnology. Anti-LSD1 (#2184), cdc37 (#3604), Histone H3 (#4499), p62/Sqstm1 (#5114), NBR1 (#9891) antibodies were purchased from Cell Signaling Technology. Anti-MSS1/PSMC2 (#14905-1-AP), Rad23A (#51033-1-AP), Rad23B (#12121-1-AP), and PDLIM7 (#10221-1-AP) antibodies were purchased from Proteintech. Anti-Ubiquilin1 (#SAB2102632) antibody was purchased from SIGMA-ALDRICH. Anti-Ubiquilin2 (#MABN763) antibody was purchased from MerckMillipore. Anti-PDLIM2 antibody to detect murine PDLIM2 (**Figures 5C,E**) was previously described as SLIM antiserum (7). Anti-PDLIM2 antibody (#ab119243) to detect human PDLIM2 (Supplementary Figure 8) was purchased from Abcam. HRP-conjugated anti-c-Myc antibody (M047-7), anti-c-Myc antibody-conjugated agarose (M047-8), anti-HA antibody (M180-3), and anti-LC3 antibody (PM036) were purchased from MBL. DDDDK-tagged protein purification gel (MBL, #3328) was used as anti-DYKDDDDK antibody-conjugated agarose. Anti-ubiquitin antibody (clone FK-2; BML-PW8810) was purchased from Enzo Life Sciences. HRP-goat anti-rabbit IgG (656120) was from Zymed and HRP-conjugated sheep anti-mouse IgG (NA931) was from GE Healthcare and used as secondary antibodies.

### Cells, Transfection, Reporter Assay

**Figure 1:**
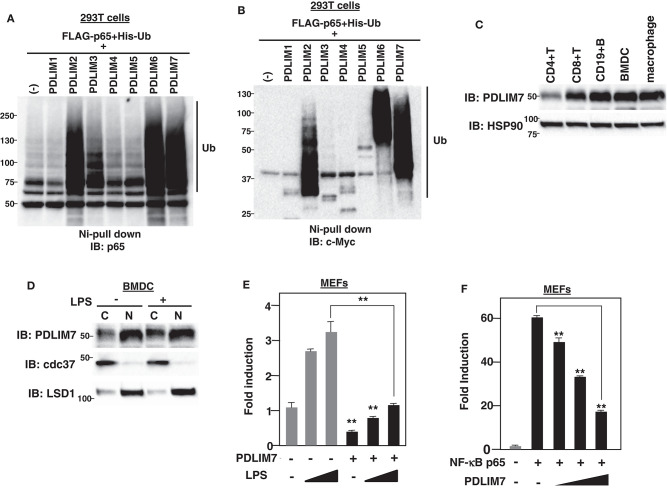
PDLIM7 is a ubiquitin E3 ligase that inhibits NF-**κ**B activation. **(A,B)** Ubiquitination assay for p65 in 293T cells transfected with plasmids encoding histidine-tagged ubiquitin (His-Ub), p65 and PDLIM1-7 or empty vector (–). Ubiquitinated proteins were purified with Ni beads. Polyubiquitination of p65 **(A)** or autoubiquitination of PDLIM1-7 **(B)** were analyzed by immunoblot (IB) with anti-p65 or anti-c-Myc antibody, respectively. Western blots are representative of two independent experiments. **(C)** Western blot analysis of PDLIM7 expression in primary immune cells, including splenic CD4+T, CD8+T, CD19+B cells, Flt3L-differentiated bone marrow derived dendritic cells (BMDC) and peritoneal macrophages. Whole cell lysates were subjected to immunoblot with anti-PDLIM7 and HSP90 antibodies. Western blots are representative of four independent experiments. **(D)** Flt3L-defferentiated bone-marrow derived dendritic cells (BMDC) were stimulated without or with LPS (100 ng/ml) for 5 h and fractionated into cytoplasmic (C) and nuclear (N) fractions, which were then analyzed by Western blotting with anti-PDLIM7 antibody. The purity of the fractionations was confirmed by blotting with anti-cdc37 (cytoplasm) or anti-LSD1 (nucleus) antibody. Western blots are representative of three independent experiments. **(E)** Luciferase activity in MEFs transfected with an ELAM−1 luciferase reporter construct (ELAM-1-luc) and pRL-Null renilla constructs along with or without a plasmid encoding PDLIM7, then left untreated or treated with LPS (2 or 10 ng/ml) for 5 h. Data are representative of four independent experiments. **(F)** Luciferase activity in MEFs transfected with ELAM-1-luc and pRL-Null with or without plasmids encoding NF-**κ**B p65 in the absence or presence of increasing amounts (wedge) of PDLIM7. Data are representative of four independent experiments. Data in **(E,F)** are means ± SD. **P < 0.01.

We basically used HEK293T cells for coimmunoprecipitation and ubiquitination experiments, mouse embryonic fibroblasts (MEFs) for luciferase assay and NIH3T3 cells for the experiments that analyze p65 degradation. HEK293T cells were cultured in DMEM supplemented with 10% FCS. MEFs were prepared from 13.5 dpc embryos. Embryos were minced and incubated in 2.5% Trypsin, 0.5 mM EDTA for 30 min at 37°C. Collected cells were then cultured in DMEM supplemented with 10% FCS and immortalized by repetitive passage. NIH3T3 cells were cultured in DMEM supplemented with 10% calf serum. GM-CSF-differentiated bone marrow cells (GM-CSF-BMCs) were prepared by culture of bone marrow cells for 5 days with murine GM-CSF (10 ng/ml) (**Figures 3A–D**, **4G**, **7A–C**; Supplementary Figures 3, 7). Flt3L-differentiated bone marrow derived dendritic cells (BMDC) were prepared by culture of bone marrow cells for 8 days with human Flt3L (50 ng/ml) (Figures 1C,D, **6B**). CD4+T, CD8+T and CD19+B cells were purified from spleen using CD4 (#130-117-043), CD8 (#130-117-128), CD19 (#130-052-201) MicroBeads, respectively (Miltenyi Biotech). For peritoneal macrophages, resident peritoneal cells were collected from the peritoneal cavity by washing the PBS and incubated on culture dish for 3 h. The adherent cells were then collected and used as macrophages. For transfections, cells were transiently transfected with Effectene (QIAGEN). For the transfection of 293T cells, cells were seeded on 600 mm diameter culture dishes (2 × 10^6^ cells/dish) and after 20 h were transfected with total 2 μg of plasmids (The dishes should be 50% confluent on the day of transfection). Cells were cultured for additional 24 h without changing the media, then lysed and subjected to immunoprecipitation assay. For the transfection of NIH3T3 cells, cells were seeded on 350 mm diameter culture dishes (3.8 × 10^5^ cells/dish) and after 20 h were transfected with total 1 μg of plasmids. Cells were cultured for additional 24 h without changing the media, then fractionated and subjected to immunoblot analysis. For the reporter assay, MEFs were seeded on 350 mm diameter culture dishes (1.8 × 10^5^ cells/dish) and after 20 h were transfected with the ELAM−1 luciferase and pRL-Null renilla constructs without or with the PDLIM7 vector, or with the ELAM−1 luciferase and the pGL4.32-Null or pRL-Null renilla constructs, and expression plasmids encoding FLAG-p65 plus wild-type PDLIM7 or the ΔLIM3 mutant. Total amounts of transfected DNA were kept constant to 1 μg/dish by supplementing with control plasmids. Cells were cultured for additional 24 h without changing the media, and then left untreated or stimulated for 5 h with LPS. Luciferase activity was measured according to the manufacturer's protocol in the Dual Luciferase Reporter System (Promega).

### Subcellular Fractionation, Immunoprecipitation and Immunoblot Analysis

All lysis buffers used for immunoblot analysis contained a protease inhibitor “cocktail” (Roche). Cytoplasmic, nuclear soluble, and nuclear insoluble extracts were prepared as previously described (9). The purity of each fraction was confirmed by blotting with anti-cdc37 (for cytoplasm), anti-LSD1 (for nuclear soluble fraction), and anti-Lamin B or Histone H3 (for nuclear insoluble fraction). Whole cell extracts were prepared by lysing cells in 50 mM Tris pH 8.0, 1% Triton X-100, 0.5 mM EDTA, 150 mM NaCl, 50 mM sodium fluoride (Figure 1C) or RIPA buffer (25 mM Tris pH 7.6, 150 mM NaCl, 1% NP-40, 1% sodium deoxycholate 0.1% SDS) (Supplementary Figures 4, 7). For immunoprecipitation, cells were lysed in RIPA buffer, incubated with anti-c-Myc or anti-DYKDDDDK antibody-conjugated agarose beads (MBL) overnight at 4°C, and washed four times by RIPA buffer. Immunopresipitated complexes were then eluted by boiling and subjected to immunoblot analysis. Samples were separated on 7.5 or 10% SDS-polyacrylamide gel (WAKO) and transferred onto nitrocellulose membrane (Thermo Fisher Scientific). Membranes were blocked with 5% milk dissolved in TBST (Nacalai tesque) or Bullet Blocking One (Nacalai tesque) and incubated sequentially with primary and secondary antibodies diluted in TBST or Can get Signal immunoreaction enhancer solution (TOYOBO). Membranes were then incubated with Chemi-Lumi One Super, ECL (Nacalai tesque) and detected using ChemiDoc XRS Plus System (Bio-Rad).

### Ubiquitination Assay

293T cells were transfected with expression plasmids encoding FLAG-tagged p65, His-tagged ubiquitin, c-Myc-PDLIM7 and PDLIM2 as indicated. His-tagged proteins were purified as previously described (15). Briefly, cells were lysed under denaturing condition with a buffer containing 6 M guanidium-HCl. Extracts were incubated with His60 Nickel Superflow Resin (TAKARA BIO INC.) for 2.5 h and then washed with buffer containing 25 mM Tris pH 6.8, 20 mM imidazole. Purified proteins were subjected to immunoblot with anti-p65, anti-c-Myc antibody or PDLIM2 antiserum.

### Small Interfering RNA (siRNA)

For 293T cells, cells (2 × 10^6^ cells) were transfected with 5 μl of 20 mM siRNA on 600 mm culture dishes by Lipofectamine RNAiMAX (Invitrogen) and after 20 h were further transfected with total 2 μg of plasmids. One day after final transfection, cells were lysed by RIPA buffer and subjected to immunoprecipitation assay. For BMCs, cells (6 × 10^6^ cells) were transfected with 15 μl of 20 mM siRNA by the Neon Transfection System (Invitrogen). Forty-eight hours after transfection, cells were harvested and stimulated with LPS as indicated and subjected to real-time PCR analysis or stimulated with LPS for 24 h and the amount of IL-6 in the supernatants was determined by ELISA (R&D Systems, #DY406). Alternatively, cells were stimulated with LPS for 1 h, fractionated and then analyzed by Western blotting. The siRNA for human PDLIM7, human p62/Sqstm1, and human PDLIM2 were purchased from Ambion (Silencer Select); human *PDLIM7*; 5′-AGGAGCACCUGAAGAAAUC-3′, human *SQSTM/p62*; 5′- CUUCCGAAUCUACAUUAAA-3′, human *PDLIM2*; 5′-GCAGGAAAAUCGCGAGGGA-3′, and control siRNA (4390843). The following siRNAs were purchased from Invitrogen (Stealth RNAi); murine *Pdlim7*; 5′-GGGUACUGAAUAUUGACGGUGAGAA3′, murine *Pdlim2*; 5′-CAGAGAUUUCCACACACCCAUCAUU-3′, murine *p62/Sqstm1*; 5′- GAUGACAUCUUCCGCAUCUACAUUA-3′, and control siRNA (12935-300).

### Real-Time Reverse Transcriptase-Polymerase Chain Reaction (RT-PCR) Analysis

Total RNA was prepared by an RNAeasy micro kit (Qiagen) and cDNA was generated with a PrimeScript RT reagent kit (TAKARA BIO INC.). Quantitative real-time PCR analyses were done using StepOnePlus Real-Time PCR System (Applied Biosystems). For PCR reaction, primer sets and probes for mouse *Il-6* (Mm00446190), *Il-12b* (Mm00434174), *Tnf* α (Mm00443258), *Csf3* (Mm00438334), *Cxcl2* (Mm00436450), *Cxcl10* (Mm00445235), *Pdlim6* (Mm00522021), *Pdlim7* (Mm00482816), *Sqstm1* (Mm00448091), and 18S rRNA (4319413E) from TaqMan Gene Expression Assay were used with Taqman Fast Advanced Master Mix (Applied Biosystems). Data were normalized to 18S rRNA and relative expression was calculated by ΔΔCt method.

### Mice

Mice were purchased from CREA Japan, Inc. All mice used were between 7 and 8 weeks of age. All experiments were approved by the Institutional Animal Care and Use Committee (IACUC) of RIKEN Yokohama Branch and conducted in accordance with the committee's guidelines.

### Statistical Analyses

All the experiments were repeated as indicated in the figure legends. The statistical significance was analyzed by Student's *t*-test. Data are shown as the mean values ± the standard deviation of the mean (SD).

## Results

### PDLIM7 Is a Ubiquitin E3 Ligase That Inhibits NF-κB Activation

Based on the structural similarity to a “really interesting new gene” (RING) finger domain, a well-known domain responsible for polyubiquitination (16), we have previously shown that the LIM domain also has ubiquitin E3 ligase activity (7). In this study, we sought to identify additional PDZ-LIM proteins, apart from PDLIM2, involved in the degradation of NF-**κ**B as ubiquitin E3 ligases in dendritic cells. We first transfected human embryonic kidney (HEK) 293T cells with expression plasmids encoding p65, PDLIM1-7, and His-tagged ubiquitin. His-tagged proteins were purified by nickel-pull down, and polyubiquitination of p65 was detected with a p65 Ab. We had predicted that all the other PDZ-LIM proteins would be ubiquitin E3 ligases, but, unexpectedly, only PDLIM2, PDLIM6, and PDLIM7 promoted polyubiquitination of p65 (Figure 1A) or of PDLIM2, 6 and 7 themselves (Figure 1B), indicating that only these three members of the PDZ-LIM protein subfamily have ubiquitin E3 ligase activity. We then examined the expression of PDLIM6 and PDLIM7; as with PDLIM2 (7), PDLIM7 is ubiquitously expressed in all immune cells tested, including dendritic cells (Figure 1C), while PDLIM6 is expressed at high levels in skeletal muscle, as previously reported (17), but only at very low levels in immune cells (Supplementary Figure 1). These data suggested that, among PDZ-LIM protein family members, only PDLIM2 and PDLIM7 are ubiquitin E3 ligases that mediate polyubiquitination of p65 in dendritic cells. These observations prompted us to select PDLIM7 for further analysis. Since both PDZ and LIM domains function as a protein-protein interaction domain, previous studies of PDLIM7 have been focused on the identification of its binding partners (10, 12) and have shown that PDLIM7 can associate with the insulin receptor (18), the receptor tyrosine kinase RET (19), and protein kinase C (PKC) (20) via its LIM domain, and β-tropomyosin (21) through its PDZ domain, although the biological significance of these interactions has not been elucidated. Moreover, PDLIM7 deficiency in mice results in mild cardiac dysfunction and systemic thrombosis (22, 23), however, the function of PDLIM7 in the immune system remains completely unclear. We therefore investigated the role of PDLIM7 in regulating NF-**κ**B signaling as a ubiquitin E3 ligase. We first examined the subcellular localization of PDLIM7 in dendritic cells and found that it resided in both the cytoplasm and nucleus, but was predominantly located in the nucleus, and this localization was not affected by LPS stimulation (Figure 1D). We next examined the effect of PDLIM7 on TLR-induced, NF-**κ**B-dependent gene activation in a reporter assay. Mouse embryonic fibroblasts (MEFs) were transfected with a plasmid encoding a luciferase reporter regulated by NF-**κ**B and left untreated or treated with the TLR ligand LPS for 5 h and then assayed for luciferase activity. Stimulation of cells with LPS augmented luciferase reporter activity, whereas coexpression of PDLIM7 potently suppressed this LPS-induced luciferase reporter transactivation (Figure 1E). Since PDLIM7 can polyubiquitinate p65 (Figure 1A), the target of PDLIM7-mediated inhibition of NF-**κ**B signaling is likely to be p65. We therefore examined the effect of PDLIM7 on p65-mediated gene activation in a reporter assay. We cotransfected MEFs with p65, to activate the NF-**κ**B luciferase construct, together with or without PDLIM7 to test if PDLIM7 could inhibit gene activation by p65. As shown in Figure 1F, PDLIM7 markedly inhibited p65-mediated transactivation of the reporter in a dose-dependent manner. These data suggest that PDLIM7 is a ubiquitin E3 ligase that inhibits p65-mediated NF-**κ**B signaling.

### PDLIM7 Promotes Polyubiquitination and Degradation of p65 via Its LIM Domain

**Figure 2:**
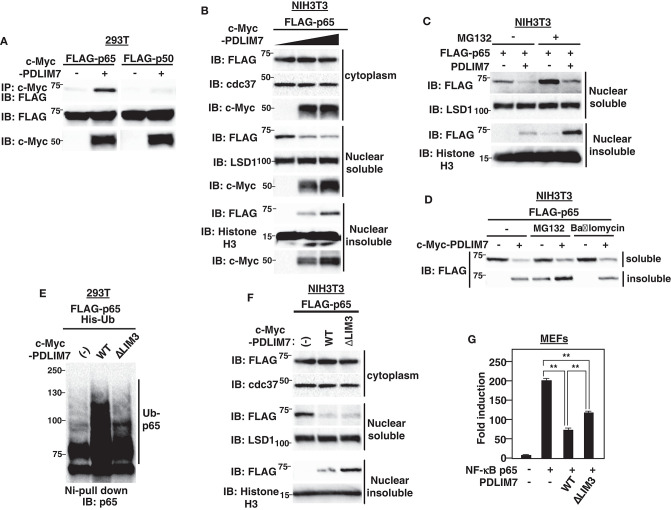
PDLIM7 promotes p65 degradation in a LIM3 domain-dependent manner. **(A)** PDLIM7 interacts with p65 but not with p50 subunit of NF-**κ**B. 293T cells were transfected with a FLAG-tagged p65 or p50 expression plasmid along with or without PDLIM7. Whole cell extracts were immunoprecipitated with anti-c-Myc and immunoblotted with anti-FLAG. Western blots are representative of four independent experiments. **(B)** Effect of PDLIM7 on cytoplasmic, soluble and insoluble nuclear p65 in NIH3T3 cells transfected with plasmids encoding FLAG-tagged p65 and increasing amounts of c-Myc-tagged PDLIM7. Cells were subjected to fractionation and analyzed with the indicated antibody. Western blots are representative of three independent experiments. **(C)** Western blot analysis for soluble and insoluble nuclear p65 in NIH3T3 cells transfected with plasmids encoding p65 without or with PDLIM7, then left untreated or treated for 4 h with MG132 (10 μM) and analyzed as in **(B)**. **(D)** NIH3T3 cells were transfected with FLAG-p65 along with or without c-Myc-PDLIM7, left untreated or treated for 4 h with MG132 (10 μM) or bafilomycin (100 μM). Cells were subjected to fractionation and analyzed with anti-FLAG antibody. Western blots are representative of three independent experiments. **(E)** Ubiquitination assay for p65 in 293T cells cotransfected with plasmids encoding His-tagged ubiquitin (His-Ub) and p65, together without or with wild-type or ΔLIM3 PDLIM7 and analyzed as in Figure 1A. Western blots are representative of three independent experiments. **(F)** Effect of ΔLIM3 PDLIM7 on cytoplasmic, soluble and insoluble nuclear p65 in NIH3T3 cells transfected with plasmids encoding p65, together without or with wild-type or ΔLIM3 PDLIM7 and analyzed as in **(B)**. Western blots are representative of five independent experiments. **(G)** Luciferase activity in MEFs transfected with an ELAM-1-luc and pGL4.32-Null renilla construct with or without plasmids encoding p65 in the absence or presence of expression plasmids encoding wild-type or ΔLIM3 PDLIM7. Data are representative of three independent experiments. Shown are the mean values ± SD. **P < 0.01.

We next investigated the mechanisms by which PDLIM7 negatively regulates p65 activation. We first tested if PDLIM7 bound to p65. 293T cells were transiently transfected with expression plasmids encoding c-Myc-tagged PDLIM7 with or without FLAG-tagged p65 or p50. PDLIM7 was co-immunoprecipitated with p65 but not with p50 (Figure 2A), suggesting that PDLIM7 selectively binds to the p65 component of NF-**κ**B. To examine the effects of PDLIM7 on p65 protein level, we next measured p65 in cytoplasmic, soluble and insoluble nuclear fractions of NIH3T3 cells transfected with FLAG-tagged p65 and PDLIM7 expression plasmids. The purity of subcellular fractions was confirmed by blotting with anti-cdc37 for cytoplasmic, anti-LSD1 for nuclear soluble and anti-Histone H3 for nuclear insoluble fractions (Supplementary Figure 2A). Note that the soluble nuclear fraction represents the sites of active transcription, while the insoluble nuclear fraction is composed of the nuclear matrix and subnuclear compartments, including PML nuclear bodies. We demonstrated in the previous paper that p65 is degraded in PML nuclear bodies where the components of proteasome are enriched (9). Cytoplasmic p65 protein expression was unaffected by PDLIM7 overexpression. In the soluble nuclear fraction, PDLIM7 markedly decreased p65 protein expression, whereas the amount of p65 protein was increased by PDLIM7 in the insoluble nuclear fraction (Figure 2B). Treatment of the cells with MG132, an inhibitor of proteasomal degradation, led to increased p65 protein in the insoluble nuclear fraction (Figure 2C). Notably, p65 in the soluble nuclear fraction, which was decreased by PDLIM7, was partially recovered by MG132 treatment. These data suggest that, similar to PDLIM2, PDLIM7 transports p65 from soluble to insoluble nuclear compartments, which correspond to PML nuclear bodies in this case, and p65 is finally degraded by the proteasome in this compartment. In contrast to the MG132 treatment, p65 accumulation in the insoluble nuclear fraction was not observed when cells were treated with bafilomycin A1, an inhibitor of V-ATPase for lysosomal degradation (Figure 2D). The effectiveness of the bafilomycin A1 treatment was confirmed by an increase in LC3-II protein levels, which is an often used marker for autophagy impairment (Supplementary Figure 2B). These data indicate that PDLIM7-mediated p65 degradation occurred in the proteasome and not in the lysosome or autophagosome.

To elucidate the function of the LIM domain of PDLIM7 in p65 ubiquitination and degradation, we next generated PDLIM7 mutants lacking PDZ or LIM domains (Supplementary Figure 2C). Although the ΔLIM3 mutant that lacks only the third LIM domain could bind to p65 (Supplementary Figure 2D), it was impaired to polyubiquitinate p65 (Figure 2E), indicating the essential role of the LIM domain in PDLIM7-mediated p65 polyubiquitination. We then transfected NIH3T3 cells with plasmids encoding wild-type or PDLIM7-ΔLIM3, together with FLAG-tagged p65 and measured p65 in cytoplasmic, soluble and insoluble nuclear fraction. Cytoplasmic p65 protein expression was unaffected by overexpression of either wild-type or PDLIM7-ΔLIM3. As expected, wild-type PDLIM7 decreased soluble p65 and increased insoluble p65 (Figure 2F). PDLIM7-ΔLIM3 decreased soluble p65 protein as with wild-type, suggesting that the LIM domain-mediated p65 polyubiquitination is not necessary for intranuclear transport of p65 from soluble to insoluble compartments. Notably, the activity of PDLIM7-ΔLIM3 to suppress p65-mediated gene activation in the luciferase assay was partially impaired due to insufficient p65 degradation (Figure 2G). However, it was not completely impaired since this mutant still had the ability to sequester p65 in a distinct intranuclear compartment where it could be separated from sites of active transcription. It is of note that insoluble p65 protein in PDLIM7-ΔLIM3 transfectants was further increased compared to that in wild-type transfectants (Figure 2F). This increase can be due to insufficient p65 degradation in the insoluble fraction, since the ability of PDLIM7-ΔLIM3 to polyubiquitinate p65 is abrogated. These data indicate the essential role of the third PDLIM7 LIM domain to promote polyubiquitination and degradation of p65.

### Enhanced p65-Mediated Inflammatory Responses Resulting From PDLIM7 Deficiency

**Figure 3:**
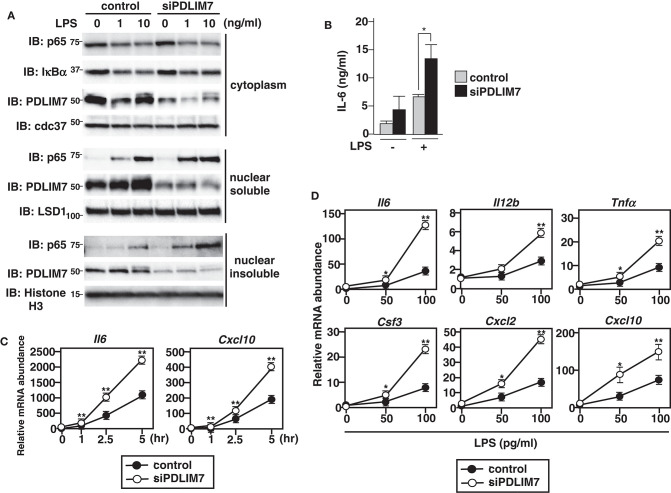
Enhanced p65 activation in PDLIM7-deficienct cells. **(A)** Immunoblot of cytoplasmic and nuclear extracts (soluble and insoluble) in GM-CSF-differentiated bone marrow cells (GM-CSF-BMCs) transfected with control or PDLIM7-specific siRNA, then left unstimulated or stimulated with the indicated concentration of LPS for 1 h and analyzed with antibodies against the indicated targets. Data are representative three independent experiments. **(B)** Enzyme-linked immunosorbent assay (ELISA) of IL-6 in culture supernatants of GM-CSF-BMCs transfected with control or PDLIM7-specific siRNA, stimulated for 24 h with LPS (0.1 ng/ml). Data are means ± SD from four independent experiments. *P < 0.05. **(C,D)** Real-time PCR analysis of proinflammatory cytokine transcript levels in GM-CSF-BMCs transfected with control or PDLIM7-specific siRNA, unstimulated or stimulated with 100 pg/ml for 1, 2.5, and 5 h **(C)** or with the indicated concentration of LPS for 5 h **(D)**. Data are representative of four **(C)** or two **(D)** independent experiments. Shown are the mean values ± SD. *P < 0.05; **P < 0.01.

To investigate the physiological roles of PDLIM7 in the regulation of TLR-mediated p65 activation, we used siRNA to knock down PDLIM7 in GM-CSF-differentiated bone marrow cells (GM-CSF-BMCs). We stimulated the cells with LPS and analyzed p65 protein in the cytoplasmic, nuclear soluble and nuclear insoluble fractions by immunoblot. The purity of subcellular fractionation in dendritic cells was confirmed by blotting with anti-cdc37 for cytoplasmic, anti-LSD1 for nuclear soluble and anti-Histone H3 for nuclear insoluble fractions (Supplementary Figure 3). Specific knockdown of PDLIM7 reduced the amount of PDLIM7 protein compared to control cells in all these fractions, and led to a marked increase in nuclear soluble and insoluble p65 protein compared to control cells, whereas the amounts of cytoplasmic p65 were not affected (Figure 3A). Notably, LPS-induced degradation of I**κ**Bα in PDLIM7 knock down cells was comparable to that in control cells. These findings indicate that LPS-induced nuclear translocation of p65 was normal in PDLIM7 knock down cells, thereby suggesting that the increased nuclear p65 protein in these cells was ascribed to impaired p65 degradation in the nucleus. Consistent with these data, PDLIM7 knockdown GM-CSF-BMCs produced two-fold more IL-6 in response to LPS than did control cells (Figure 3B). We next examined the time course of TLR-induced expression of proinflammatory cytokine genes in PDLIM7 knockdown cells. Control and PDLIM7 knockdown GM-CSF-BMCs were stimulated with LPS for 1, 2.5, and 5 h, and IL-6 and CXCL-10 mRNA expression was analyzed by real-time PCR. The expression of these proinflammatory cytokines was markedly enhanced in PDLIM7 knockdown cells at all time points (Figure 3C). Notably, this enhancement was most prominent at 5 h of LPS stimulation. We therefore stimulated control and PDLIM7 knock down GM-CSF-BMCs for 5 h with LPS and checked the expression of other TLR-inducible genes. As shown in Figure 3D, we observed a consistent two- to three-fold increase in IL-6, IL-12β, TNFα, G-CSF, CXCL-2 and CXCL-10 transcripts in response to LPS compared to control cells. These data suggest that PDLIM7 inhibits NF-**κ**B-mediated inflammatory responses by controlling nuclear, but not cytoplasmic, p65 protein levels.

### PDLIM7 and PDLIM2 Form Dimers and Synergistically Promote p65 Degradation

**Figure 4:**
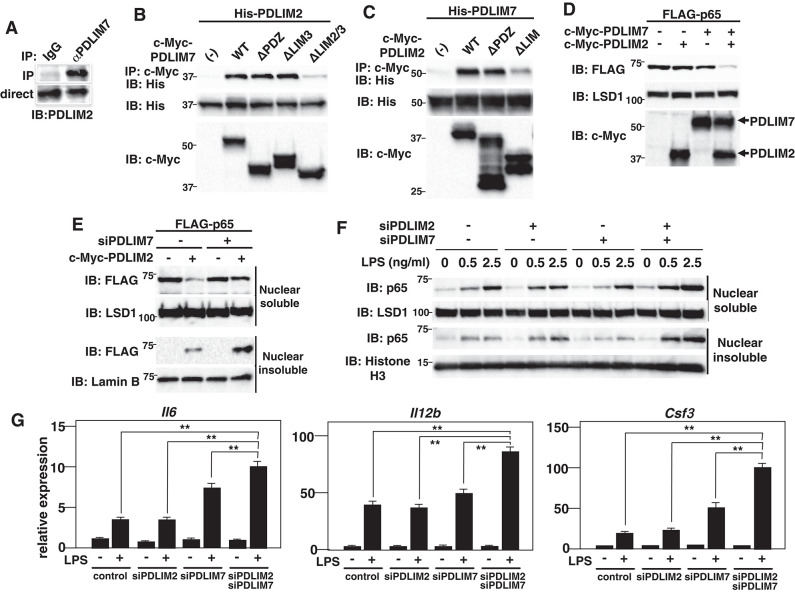
PDLIM7 and PDLIM2 form dimers and synergistically degrade p65. **(A)** PDLIM7 interacts with PDLIM2 *in vivo*. RAW264.7 cell lysates were immunoprecipitated with anti-PDLIM7 or rabbit IgG, and then blotted with anti-PDLIM2 antiserum. Western blots are representative of three independent experiments. **(B)** Critical region of PDLIM7 for interaction with PDLIM2. Immunoblot of the lysates from 293T cells transfected with His-PDLIM2 plus c-Myc-tagged wild-type or deletion mutants of PDLIM7, and immunoprecipitated with anti-c-Myc. Western blots are representative of four independent experiments. **(C)** Critical region of PDLIM2 for association with PDLIM7. Immunoblot of the lysates from 293T cells transfected with His-PDLIM7 plus c-Myc-tagged wild-type or deletion mutants of PDLIM2 and immunoprecipitated with anti-c-Myc. Western blots are representative of four independent experiments. **(D)** Synergistic effect of PDLIM7 and PDLIM2 on soluble nuclear p65 in NIH3T3 cells transfected with plasmids encoding p65, PDLIM7, or PDLIM2 in the indicated combination and analyzed with anti-FLAG antibody. Western blots are representative of three independent experiments. **(E)** Effect of PDLIM7 deficiency on soluble and insoluble nuclear p65 in 293T cells first transfected with control siRNA or PDLIM7-specific siRNA, then transfected with plasmids encoding FLAG-p65 without or with c-Myc-PDLIM7 and analyzed with anti-FLAG antibody. Western blots are representative of three independent experiments. **(F)** Immunoblot of nuclear soluble and insoluble extracts of MEFs transfected with control or PDLIM2 and/or PDLIM7-specific siRNA, stimulated with LPS for 1 h, and analyzed with the indicated antibodies. Western blots are representative of two independent experiments. **(G)** Real-time PCR analysis of proinflammatory cytokine transcript levels in GM-CSF-BMCs transfected with control or PDLIM2 and/or PDLIM7-specific siRNA, unstimulated or stimulated with LPS (0.1 ng/ml) for 5 h. Data are representative of four independent experiments. Shown are the mean values ± SD. **P < 0.01.

Several RING finger-type E3 ligases are reported to form heterodimers in which the E3 ligase activity to ubiquitinate and degrade substrate proteins is enhanced compared to the individual monomers (24). Examples of such heterodimers include Mdm2-MdmX (25–27), BRCA1-BARD1 (28, 29) and RING1B-Bmi1 (30). We therefore examined whether PDLIM7 and PDLIM2 form dimers and function synergistically to regulate p65 activation. We first examined the potential interaction of PDLIM7 and PDLIM2 in RAW264.7 cells, in which both PDLIM7 and PDLIM2 are highly expressed. Endogenous coimmunoprecipitation experiments indicated that PDLIM7 was associated with PDLIM2 *in vivo* (Figure 4A). We then transfected 293T cells with expression plasmids encoding c-Myc-tagged PDLIM7 or the PDLIM7 mutant lacking the PDZ domain (ΔPDZ), the third LIM domain (ΔLIM3) or the second and third LIM domains (ΔLIM2/3) (Supplementary Figure 2C) together without or with His-tagged PDLIM2. Whole cell extracts were subjected to immunoprecipitation with anti-c-Myc antibody and immunoblot with anti-His antibody. As shown in Figure 4B, PDLIM2 coimmunoprecipitated with wild-type, ΔPDZ and ΔLIM3 PDLIM2, but not with ΔLIM2/3, suggesting that second, but not third, LIM domain of PDLIM7 is essential for its binding to PDLIM2. We further transfected 293T cells with expression plasmids encoding c-Myc-tagged PDLIM2 or the PDLIM2 mutant lacking the PDZ domain (ΔPDZ) or LIM domain (ΔLIM) together without or with His-tagged PDLIM7. Whole cell extracts were prepared and subjected to immunoprecipitation with a c-Myc antibody and then analyzed by Western blotting with a His antibody. PDLIM7 bound to wild-type, and ΔPDZ but not to ΔLIM PDLIM2 (Figure 4C). These data suggest that PDLIM7 and PDLIM2 form dimers through the second LIM domain of PDLIM7 and the LIM domain of PDlLIM2. To examine the effects of PDLIM7 on PDLIM2-mediated p65 degradation, we next transfected NIH3T3 cells with plasmids encoding FLAG-tagged p65 and c-Myc-tagged PDLIM2 together without or with c-Myc-tagged PDLIM7 at a suboptimal concentration, in which lower amount of PDLIM7 was transfected compared to Figure 2F. While the transfection of either PDLIM2 or PDLIM7 alone minimally decreased p65 protein in these conditions, coexpression of PDLIM2 and PDLIM7 resulted in marked decrease of p65 protein in the soluble nuclear fraction (Figure 4D). We then measured p65 levels in soluble and insoluble nuclear fractions of 293T cells transfected with p65 and PDLIM2 expression plasmids in the absence or presence of PDLIM7-specific siRNA to target the endogenous transcripts. The PDLIM7 protein level was substantially reduced in cells transfected with PDLIM7-specific siRNA (Supplementary Figure 4). PDLIM7 knockdown led to increase of p65 protein, mainly in the insoluble nuclear fraction where p65 is degraded, whereas nuclear soluble p65 was partially restored by the siRNA treatment (Figure 4E). Since the degradation of polyubiquitinated p65 protein mainly occurs in insoluble nuclear compartment (9), these data indicate that PDLIM7 is required for the activity of PDLIM2 to degrade p65 protein in that compartment. To further demonstrate that PDLIM7 synergistically promotes p65 degradation with PDLIM2, we knocked down PDLIM2 and PDLIM7 in fibroblasts and measured the amount of p65 in the soluble and insoluble nuclear fractions. We found that p65 protein was increased in the soluble and insoluble nuclear fractions of LPS-stimulated cells lacking both PDLIM7 and PDLIM2 compared to stimulated control cells or in stimulated cells lacking either PDLIM7 or PDLIM2 (Figure 4F). Consistently, double knockdown of PDLIM7 and PDLIM2 in GM-CSF-BMCs resulted in augmented production of proinflammatory cytokines, IL-6, IL-12β, and G-CSF, compared to control cells or single knockdown cells (Figure 4G). These data suggest that PDLIM7 and PDLIM2 form heterodimers that synergistically promote p65 degradation and suppress inflammatory responses.

### PDLIM7 Ubiquitinates PDLIM2 and Facilitates Its Activity to Degrade p65

**Figure 5:**
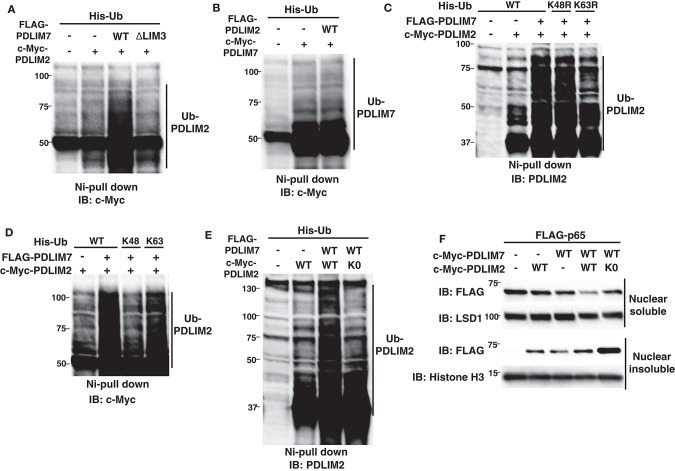
PDLIM7 ubiquitinates PDLIM2 to enhance its activity to degrade p65. **(A)** Ubiquitination assay for PDLIM2 in 293T cells cotransfected with plasmids encoding His-Ub and PDLIM2, together without or with wild-type PDLIM7 or the ΔLIM3 mutant and analyzed as in Figure 1A. Western blots are representative of four independent experiments. **(B)** Ubiquitination assay for PDLIM7 in 293T cells cotransfected with plasmids encoding His-Ub and PDLIM7, together without or with PDLIM2. Western blots are representative of three independent experiments. **(C)** Ubiquitination assay for PDLIM2 in 293T cells cotransfected with plasmids encoding His-tagged wild-type Ub or K48R or K63R mutants, in which the lysine at K48 or K63 was mutated to arginine, respectively, together with c-Myc-PDLIM2 and/or FLAG-PDLIM7. Western blots are representative of two independent experiments. **(D)** Ubiquitination assay for PDLIM2 in 293T cells cotransfected with plasmids encoding His-tagged wild-type Ub or K48 or K63 mutants (with lysine only at position 48 or 63, respectively), together with c-Myc-PDLIM2 and/or FLAG-PDLIM7. Western blots are representative of three independent experiments. **(E)** Ubiquitination assay for PDLIM2 in 293T cells cotransfected with plasmids encoding His-Ub and wild-type PDLIM2 or the K0 mutant, in which all nine lysine residues were mutated to arginine, together without or with PDLIM7. Western blots are representative of seven independent experiments. **(F)** Effect of the K0 PDLIM2 mutant on soluble and insoluble nuclear p65 in NIH3T3 cells transfected with plasmids encoding FLAG-p65 and PDLIM7, together without or with wild-type PDLIM2 or the K0 mutant. Cells were subjected to fractionation and analyzed by anti-FLAG antibody. Western blots are representative of eight independent experiments.

We next examined the molecular mechanisms by which PDLIM7 promotes PDLIM2-mediated p65 degradation. In other E3 heterodimers, such as Mdm2-MdmX and BRCA1-BARD1, ubiquitin E3 ligases in the complex can be autoubiquitinated or trans-ubiquitinated by their partners, which enhances E3 ligase activity of the complex (26, 27, 29). We have therefore examined if the ubiquitination of PDLIM2 or PDLIM7 was increased in the presence of PDLIM7 or PDLIM2, respectively. We found that PDLIM7 expression substantially enhanced PDLIM2 ubiquitination (Figure 5A). Notably, PDLIM2 ubiquitination was not enhanced when expressed with the ΔLIM3 PDLIM7 mutant (Figure 5A), although this mutant could still bind to PDLIM2 (Figure 4B). Since the LIM domain has ubiquitin E3 ligase activity, these data suggested that PDLIM2 was likely to be polyubiquitinated by PDLIM7 and not autoubiquitinated. In contrast, PDLIM2 could not promote polyubiquitination of PDLIM7 (Figure 5B). We then analyzed the type of ubiquitin chain linkage generated by PDLIM7-mediated PDLIM2 ubiquitination. We found that this ubiquitination was abrogated with the ubiquitin mutant in which a lysine at position 63 (K63R), but not at position 48 (K48R), was mutated to arginine (Figure 5C). Moreover, PDLIM7 promoted PDLIM2 ubiquitination only when Ub-K63 mutant (with lysine only at position 63), but not Ub-K48 mutant, was coexpressed (Figure 5D), indicating that PDLIM7 ubiquitinates PDLIM2 through ubiquitin chain K63. To determine if PDLIM2 polyubiquitination affects its activity to degrade p65, we next generated a PDLIM2 mutant lacking all nine lysine residues (PDLIM2-K0). We first confirmed that PDLIM7 could not polyubiquitinate PDLIM2-K0 (Figure 5E), although this PDLIM2 mutant could still bind to PDLIM7 (Supplementary Figure 5). We then transfected NIH3T3 cells with FLAG-tagged p65 and c-Myc-tagged wild-type or K0 PDLIM2 mutants together without or with c-Myc-tagged PDLIM7 and observed that p65 degradation by the PDLIM2 K0 was severely impaired in both nuclear soluble and insoluble fractions, compared to wild-type PDLIM2 (Figure 5F). These data demonstrate the essential role of PDLIM7-mediated PDLIM2 polyubiquitination for the activity of PDLIM2 to degrade p65.

### p62/Sqstm1 Shuttles the Ubiquitinated PDLIM2-p65 Complex to the Proteasome

**Figure 6:**
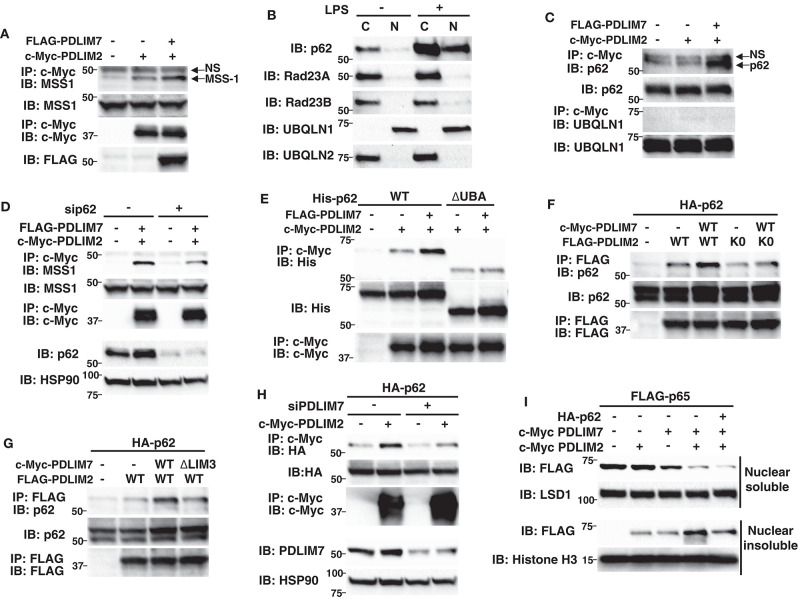
P62/Sqstm1 shuttles the ubiquitinated PDLIM2-p65 complex to the proteasome. **(A)** The effect of PDLIM7 on the interaction of PDLIM2 and the proteasome in whole cell extracts of 293T cells transfected with c-Myc-PDLIM2 and/or FLAG-PDLIM7, then immunoprecipitated with anti-c-Myc, followed by immunoblot with anti-MSS1, the Rpt1/S7 subunit of the 19S proteasome. The whole cell lysates were immunoblotted with anti-MMS1; NS, non-specific band. Western blots are representative of four independent experiments. **(B)** Expression of shuttling factors in cytoplasmic and nuclear extracts of BMDC stimulated without or with LPS (100 ng/ml) for 5 h. Western blots are representative of three independent experiments. **(C)** 293T cells were transfected with c-Myc-PDLIM2 and/or FLAG-PDLIM7, then immunoprecipitated with anti-c-Myc, followed by immunoblot with anti-p62/Sqstm1 or anti-UBQLN1; NS, non-specific band. Western blots are representative of three independent experiments. **(D)** 293T cells were first transfected with control or p62-specific siRNA, then transfected with c-Myc-PDLIM2 and/or FLAG-PDLIM7, then immunoprecipitated with anti-c-Myc, followed by immunoblot with anti-MSS1. Western blots are representative of three independent experiments. **(E)** 293T cells were cotransfected with c-Myc-PDLIM2 and FLAG-PDLIM7 together with His-p62 or its mutant lacking the UBA domain (ΔUBA) then immunoprecipitated with anti-c-Myc, followed by immunoblot with anti-His. Western blots are representative of two independent experiments. **(F)** 293T cells were cotransfected with HA-p62 and c-Myc-PDLIM7, together without or with FLAG- wild-type PDLIM2 or the K0 mutant, then immunoprecipitated with anti-FLAG, followed by immunoblot with anti-p62. Western blots are representative of five independent experiments. **(G)** 293T cells were cotransfected with HA-p62 and FLAG-PDLIM2, together without or with wild-type c-Myc-PDLIM7 or the ΔLIM3 mutant, then immunoprecipitated with anti-FLAG, followed by immunoblot with anti-p62. Western blots are representative of three independent experiments. **(H)** 293T cells were first transfected with control or PDLIM7-specific siRNA, then transfected with HA-p62 together without or with c-Myc-PDLIM2, then immunoprecipitated with anti-c-Myc, followed by immunoblot with anti-HA. Western blots are representative of three independent experiments. **(I)** Effect of p62 on soluble and insoluble nuclear p65 in NIH3T3 cells transfected with plasmids encoding FLAG-p65, c-Myc-tagged PDLIM7 and PDLIM2, together without or with p62. Cells were subjected to fractionation and analyzed by anti-FLAG antibody. Western blots are representative of three independent experiments.

We next investigated the molecular mechanisms by which PDLIM7-mediated PDLIM2 polyubiquitination affected the activity of PDLIM2 to degrade p65. We first asked if the activity of PDLIM2 to bind to and polyubiquitinate p65 is affected by its polyubiquitination by PDLIM7 and demonstrated that the PDLIM2 K0 mutant could bind to and polyubiquitinate p65 at the same level as wild-type PDLIM2 (Supplementary Figures 6A,B). Moreover, PDLIM7 knockdown in 293T cells did not affect the association of PDLIM2 with p65 and PDLIM7-mediated p65 polyubiquitination (Supplementary Figures 6C,D). These data suggested that PDLIM7-mediated PDLIM2 polyubiquitination did not alter the ability of PDLIM2 to bind to and polyubiquitinate p65. We then thought that PDLIM7-mediated PDLIM2 ubiquitination might facilitate the trafficking of the PDLIM2/p65 complex to the proteasome. 293T cells were transfected with PDLIM2 without or with PDLIM7, and association of PDLIM2 with the MSS1, Rpt1/S7 subunit of the 19S regulatory complex of the proteasome (31) was examined. A weak interaction of PDLIM2 with endogenous MSS1 was detected when PDLIM2 was expressed, and this interaction was significantly enhanced by PDLIM7 coexpression, suggesting that PDLIM7 facilitates the shuttling of PDLIM2 to the proteasome (Figure 6A). We predicted that an adaptor protein responsible for shuttling of ubiquitinated proteins to the proteasome should be involved to link ubiquitinated PDLIM2 to the proteasome and attempted to identify this adaptor protein. To date, several shuttling factors have been identified (32, 33), including Rad23 (34) (or Rad23A/B for human), Dsk2 (35) (or Ubiquilin1/2 for human), Ddi1 (36), and p62/Sqstm1 (31, 37, 38). Since PDLIM7 is involved in nuclear, but not cytoplasmic, p65 degradation, we first examined the subcellular localization of these proteins in dendritic cells. We found that p62/Sqstm1 was only expressed in the cytoplasm without TLR stimulation, but was translocated into the nucleus, where PDLIM2 is located, after 5 h of LPS stimulation (Figure 6B). In contrast, Rad23A, Rad23B and Ubiquilin-2 were only expressed in the cytoplasm and Ubiquilin-1 was located in the nucleus without or with LPS stimulation (Figure 6B), whereas DDi1 expression was hardly detectable in dendritic cells. We therefore selected p62/Sqstm1 and Ubiquilin-1, which are expressed in the nucleus, as candidate shuttling adaptors for ubiquitinated PDLIM2, and examined their interaction with PDLIM7 and/or PDLIM2. PDLIM2 bound to endogenous p62/Sqstm1, but not Ubiquilin-1, when PDLIM7 was coexpressed (Figure 6C), which prompted us to focus on p62/Sqstm1 and investigate whether p62/Sqstm1 is involved in PDLIM7-mediated delivery of ubiquitinated PDLIM2 to the proteasome.

p62/Sqstm1 is a known autophagy adaptor protein that shuttles ubiquitinated protein aggregates to the autophagic degradation machinery by interacting with polyubiquitinated chains via its ubiquitin-associated domain (UBA) and binds to LC3, an essential component of the autophagosome membrane, through its LC3-interacting region (LIR) (39). Recently, p62/Sqstm1 was also reported to deliver ubiquitinated proteins to the proteasome (37, 38). p62/Sqstm1 interacts with K63-linked polyubiquitin chains via its C-terminal UBA domain and with the proteasomal components, including MSS1/Rpt1, via its N-terminal PB1 domain, thereby facilitating the proteasomal degradation of polyubiquitinated proteins. We first tested if the association of PDLIM2 with MSS1 was affected by p62/Sqstm1-specific siRNA knockdown, which resulted in a marked decrease in p62/Sqstm1 protein levels compared to control cells, and found that p62/Sqstm1 knockdown abrogated the binding between PDLIM2 and MSS1 (Figure 6D), suggesting that p62/Sqstm1 is essential for transport of PDLIM2 to the proteasome. Since, as described above, p62/Sqstm1 can bind to ubiquitinated proteins, we next examined the effect of PDLIM7-mediated PDLIM2 ubiquitination on the binding of p62/Sqstm1 to PDLIM2. 293T cells were transfected with p62/Sqstm1 and PDLIM2 together without or with PDLIM7 and the association of p62/Sqstm1 with PDLIM2 was examined. p62/Sqstm1 could weakly interact with PDLIM2, and this interaction was significantly enhanced by PDLIM7 coexpression, suggesting that PDLIM7 facilitates the binding of p62/Sqstm1 to PDLIM2 (Figure 6E). Notably, this enhancement was abrogated when a p62/Sqstm1 mutant lacking the ubiquitin-associated domain (ΔUBA) was used (Figure 6E), or when PDLIM2 K0, a ubiquitination defective mutant, was cotransfected (Figure 6F), or when the PDLIM7 ΔLIM3 mutant, which binds to but does not ubiquitinate PDLIM2, was coexpressed (Figure 6G). Consistently, binding of p62/Sqstm1 to PDLIM2 was markedly impaired when cells were treated with PDLIM7-specific siRNA (Figure 6H). These data suggest that p62/Sqstm1 binds to polyubiquitinated PDLIM2, which is mediated by PDLIM7.

To examine the effect of p62/Sqstm1 on PDLIM2- and PDLIM7-mediated p65 degradation, we transfected NIH3T3 cells with the p65 expression plasmid plus PDLIM2, PDLIM7 and/or p62/Sqstm1 and measured nuclear p65 protein amounts (Figure 6I). Transfection of PDLIM2 and/or PDLIM7 decreased p65 in the nuclear soluble fraction to some extent, whereas cotransfection of p62/Sqstm1 plus PDLIM2 and PDLIM7 further reduced p65 protein amounts. Transfection of PDLIM2 and/or PDLIM7 increased p65 protein in the nuclear insoluble fraction, consistent with our previous finding that PDLIM2 and PDLIM7 shuttle p65 from soluble to insoluble nuclear compartments. Notably, cotransfection of p62/Sqstm1 together with PDLIM2 and PDLIM7 strikingly reduced p65 in this fraction. Considering that degradation of polyubiquitinated p65 occurs mainly in the insoluble nuclear fraction, these data indicate that p62/Sqstm1 is essential for PDLIM2 and PDLIM7-mediated, proteasome-dependent degradation of p65 protein in that compartment.

**Figure 7:**
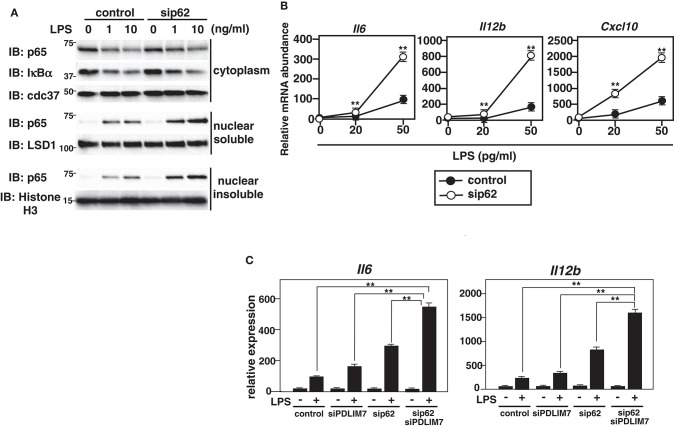
Enhanced p65 activation in p62/Sqstm1-deficient cells. **(A)** Immunoblot of cytoplasmic and nuclear extracts (soluble and insoluble) of GM-CSF-BMCs transfected with control or p62-specific siRNA, then left unstimulated or stimulated with the indicated concentrations of LPS for 1 h and analyzed with antibodies against the indicated targets. Data are representative three independent experiments. **(B)** Real-time PCR analysis of proinflammatory cytokine transcript levels in GM-CSF-BMCs transfected with control or p62-specific siRNA, unstimulated or stimulated with the indicated concentration of LPS for 5 h. Data are representative of five independent experiments. Shown are the mean values ± SD. **P < 0.01. **(C)** Real-time PCR analysis of proinflammatory cytokine transcript levels in GM-CSF-BMCs transfected with control or PDLIM7 and/or p62/Sqstm1-specific siRNA, unstimulated or stimulated with LPS (50 pg/ml) for 5 h. The samples from cells transfected with control or p62-specific siRNA in this experiment were common to the samples used in **(C)**. Data are representative of five independent experiments. Shown are the mean values ± SD. **P < 0.01.

We finally examined the physiological role of p62/Sqstm1 in the regulation of TLR-induced inflammatory responses. We knocked down p62/Sqstm1 in GM-CSF-BMCs by using siRNA, which led to a marked reduction of p62/Sqstm1 protein compared to control cells (Supplementary Figure 7). We then stimulated the cells with LPS and analyzed p65 protein amounts by immunoblot. There was more soluble and insoluble nuclear p65 in LPS-stimulated p62/Sqstm1 knockdown cells than in control cells, but the cytoplasmic p65 levels were unaffected (Figure 7A). Meanwhile, LPS-induced degradation of I**κ**Bα was comparable between control and p62/Sqstm1 knockdown cells, indicating that p65 degradation in the soluble and insoluble nuclear fraction, but not TLR signaling leading to NF-**κ**B activation, is impaired in the absence of p62/Sqstm1. Next, we examined TLR-induced production of proinflammatory cytokines in p62/Sqstm1 knockdown cells and demonstrated a consistent two- to four-fold increase in IL-6 and IL-12β transcripts in response to LPS (Figure 7B). Moreover, production of these cytokines was further enhanced in Sqstm1/p62 and PDLIM7 double knockdown cells (Figure 7C). Collectively, these data clearly suggest that p62/Sqstm1 binds to the complex of PDLIM7-dependently polyubiquitinated PDLIM2 and p65 and targets it to the proteasome, thereby facilitating p65 degradation and suppressing NF-**κ**B-mediated inflammatory responses.

## Discussion

NF-**κ**B activation in dendritic cells is essential for early phase host defense by inducing inflammatory responses and eliminating invading microbial pathogens. However, excessive NF-**κ**B activation may contribute to the pathogenesis of human autoimmune diseases (3, 4). NF-**κ**B activation must therefore be tightly regulated to prevent the onset of these diseases. One well-known mechanism to terminate NF-**κ**B activation is ubiquitin/proteasome-dependent degradation of the p65 subunit. To date, several ubiquitin E3 ligases responsible for proteasomal degradation of p65 have been reported, including SOCS1 (suppressor of cytokine signaling 1) (40), COMMD1 (COMM domain containing 1) (41), ING4 (inhibitor of growth protein 4) (42) and PPARγ (peroxisome proliferator activated receptor-γ) (43). PPARγ has RING finger domain, whereas ING4 contains PHD domain, a subtype of RING domain. On the other hand, SOCS1 and COMMD1 form a complex with Rbx1, a RING-type E3 ligase. We have previously demonstrated that PDLIM2 functions as a nuclear ubiquitin E3 ligase for p65 through its LIM domain (9) and demonstrated for the first time that the LIM domain has ubiquitin E3 ligase activity (7). PDLIM2 belongs to a large family of LIM proteins (10) as a member of the seven member PDZ-LIM protein subfamily, which is characterized by the presence of both PDZ and LIM domains (11). In this study, we investigated the role of this subfamily as ubiquitin E3 ligases in the regulation of NF-**κ**B activation. Interestingly, although all seven PDZ/LIM proteins have one or three LIM domains, only PDLIM2, 6 and 7 have E3 ligase activity and can polyubiquitinate p65. Given that PDLIM6 expression is barely detectable in dendritic cells, only PDLIM2 and PDLIM7, among PDZ/LIM proteins, are the ubiquitin E3 ligases that function in dendritic cells. The RING finger and LIM domains are thought to participate in binding to the E2 component of the ubiquitination reaction. Although LIM domains of all seven PDZ/LIM proteins contain eight conserved cysteine and histidine residues, their homology to each other in the other regions of the LIM domains is very low. Possibly because of structural differences resulting from these differences in amino acid sequence, only the LIM domains of PDLIM2, 6 and 7, but not PDLIM1, 3, 4, 5, can bind to E2 and have ubiquitin E3 ligase activity.

To date, several adaptor proteins have been identified that shuttle ubiquitinated protein substrates to the proteasome for degradation (32, 33). For example, Rad23 (34), Dsk2 (35), and Ddi1 (36) contain both a UBA domain for binding to the ubiquitin chains and a ubiquitin-like domain (UBL) for interacting with the proteasome. On the other hand, p62/Sqstm1, NBR1, NDP52, and optineurin were originally identified as ubiquitin-binding autophagy receptors that contain both UBA and LIR domains and link ubiquitinated proteins to autophagic degradation in the selective autophagy pathway (44). Among these proteins, p62/Sqstm1 and NBR1 also have a PB1 domain for associating with the proteasome, and p62/Sqstm1 was recently shown to target ubiquitinated proteins for proteasomal degradation (37, 38). Thus, p62/Sqstm1 mediates the delivery and degradation of ubiquitinated proteins by both proteasome- and autophagy-dependent pathways. Recent reports demonstrated that the p62/Sqstm1-mediated autophagy pathway suppressed inflammasome-dependent IL-1β-production and subsequent inflammatory responses by promoting removal of damaged mitochondria, which release inflammasome activators and induce NLRP3-inflammasome-dependent caspase-1 activation to induce IL-1β maturation (45, 46). However, the p62/Sqstm1-mediated autophagy pathway did not affect NF-**κ**B activation, since inflammasome-dependent IL-1β production, but not IL-1β mRNA expression, was augmented in p62/Sqstm1-deficient macrophages. In this study, we demonstrate that PDLIM7 cooperates with p62/Sqstm1 to inhibit NF-**κ**B-mediated gene activation by promoting PDLIM2-mediated proteasomal degradation of the p65 subunit of NF-**κ**B, mainly by the experiments that overexpress PDLIM7, PDLIM2 and p62/Sqstm1 proteins together with p65. Moreover, the finding that PDLIM7 and/or p62/Sqstm1 deficiency in bone marrow-derived cells resulted in augmented production of LPS-induced proinflammatory cytokines clarified the physiological relevance of these factors to the regulation of NF-**κ**B-mediated inflammatory responses.

**Figure 8:**
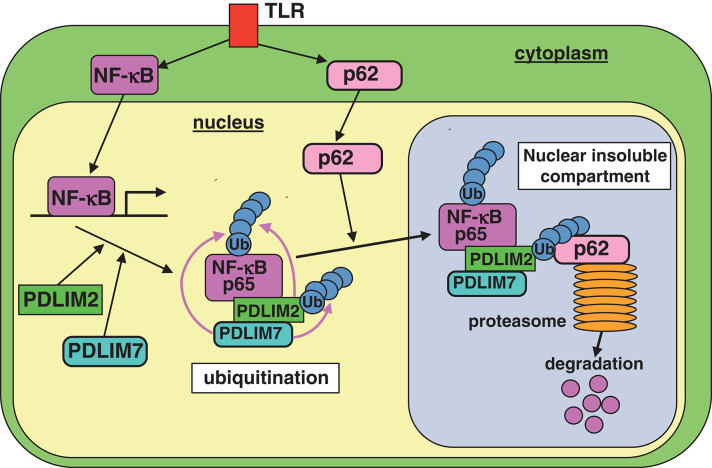
A model of the proposed molecular mechanisms by which PDLIM7 promotes PDLIM2 and p62/Sqstm1-mediated degradation of p65. First, PDLIM7 directly polyubiquitinates p65 and promotes its proteasomal degradation. Second, PDLIM7 also polyubiquitinates PDLIM2 and facilitates the p62/Sqstm1-dependent delivery of the ubiquitinated PDLIM2/p65 complex to the proteasome for p65 degradation.

We found that PDLIM7 promoted p65 degradation by two mechanisms. First, PDLIM7 directly binds to p65 and promotes its polyubiquitination and proteasomal degradation. Second, PDLIM7 also binds to PDLIM2 to form a dimer that promotes K63-linked polyubiquitination of PDLIM2 and facilitates PDLIM2-mediated p65 degradation by the proteasome, cooperatively with p62/Sqstm1. In this pathway, polyubiquitinated PDLIM2 binds to the UBA domain of p62/Sqstm1 and then p62/Sqstm1 interacts with the proteasome via its PB1 domain, thereby facilitating the delivery of the polyubiquitinated PDLIM2-NF-**κ**B complex to the proteasome (Figure 8). Notably, PDLIM7-dependent, p62/Sqstm1-mediated p65 degradation does not occur in the autophagosome, since PDLIM7-mediated p65 degradation was abrogated by MG132, an inhibitor of proteasomal degradation, but not by bafilomycin A1, which suppresses lysosomal degradation in autophagy. These findings collectively suggest that p62/Sqstm1 negatively regulates inflammatory responses by two distinct pathways, autophagy-dependent suppression of inflammasome-mediated IL-1β production and proteasome-dependent inhibition of NF-**κ**B-mediated pro-inflammatory cytokine gene expression.

Several ubiquitin E3 ligases have been reported to form heterodimers, e.g., Mdm2-MdmX (or Hdm2-HdmX in human), BRCA1-BARD1 and RING1B-Bmi1, and are therefore called heterodimeric E3 ligases (24). These ubiquitin E3 ligases tend to be autoubiquitinated or trans-ubiquitinated by their partners, which enhances E3 ligase activity of the complex. For example, although MdmX itself has little, if any, E3 ligase activity, MdmX enhances autoubiquitination of Mdm2 and promotes its ubiquitin ligase activity by facilitating binding to UbcH5c, an E2 enzyme essential for p53 ubiquitination, thereby facilitating Mdm2-mediated polyubiquitination and degradation of p53 (25–27). On the other hand, BRCA1 and BARD1 polyubiquitinate each other and this mutual ubiquitination synergistically enhances their E3 ligase activity, although the exact mechanism by which this mutual polyubiquitination facilitates the degradation of their substrates is unknown (28, 29). We have demonstrated that PDLIM7 polyubiquitinates, not only p65, but also PDLIM2, and enhances the activity of PDLIM2 to degrade p65 by a novel mechanism. K63-linked polyubiquitin chains of PDLIM2 bind to p62/Sqstm1, p62/Sqstm1 then interacts with MSS1/Rpt1, a component of the proteasome, and facilitates the delivery of PDLIM2-p65 complex to the proteasome for p65 degradation.

We could not completely determine whether PDLIM7 directly ubiquitinates PDLIM2 or enhances its autoubiquitination. However, since the ΔLIM3 mutant of PDLIM7, which impairs ubiquitin ligase activity but still binds to PDLIM2, failed to polyubiquitinate PDLIM2, PDLIM7 itself likely ubiquitinates PDLIM2. Moreover, given that PDLIM2 associates with PDLIM7, it is possible that the effect of PDLIM7 on p65 polyubiquitination might be ascribed to the indirect effect of associating PDLIM2. However, we consider this unlikely because we could not detect PDLIM2 expression in 293T cells, in which we overexpressed PDLIM7 to examine p65 polyubiquitination (Supplementary Figure 8A). Moreover, PDLIM7 could still promote p65 polyubiquitination, even when possible minimal expression of PDLIM2 in 293T cells was eliminated by human PDLIM2-specific siRNA (Supplementary Figure 8B). To evaluate the efficiency of this siRNA to reduce PDLIM2 mRNA levels, we showed the level reduced to 12.8 ± 1.1% of control in A549 cells, which highly express PDLIM2. Thus, the potential amount of PDLIM2 mRNA remaining in siRNA-treated 293T cells, in which PDLIM2 expression is barely detectable in the first place, is likely to be minuscule or non-existent. These data suggest that PDLIM7 can polyubiquitinate p65 in the absence of PDLIM2.

Constitutive activation of NF-**κ**B is reported at sites of inflammation in human autoimmune diseases, such as rheumatoid arthritis (4). It is also reported that the accumulation of protein aggregates in neurons, possibly due to inappropriate degradation in the proteasome or by autophagy, may cause neurodegenerative disorders, including Alzheimer's and Parkinson's diseases (47). We therefore speculate that dysfunction of the proteolytic system in inflammation-related cells, such as dendritic cells, fibroblasts and vascular endothelial cells, may result in persistent inflammation and the accumulation of ubiquitinated protein aggregates, which may cause degeneration of these cells. This pathology might be associated with the fibrinoid degeneration observed in autoimmune systemic vasculitis. Taken together, the PDLIM7/PDLIM2/p62-mediated degradation pathway to terminate p65 activation may prevent the onset of these autoimmune diseases and could be a novel molecular target for the treatment of autoimmune diseases.

## Data Availability Statement

All datasets generated for this study are included in the article/Supplementary Material.

## Ethics Statement

The animal study was reviewed and approved by the Institutional Animal Care and Use Committee (IACUC) of RIKEN Yokohama Branch, Japan.

## Author Contributions

AJ, AS, and AO performed experiments. TK contributed to discussion and manuscript writing. TT directed the project, performed experiments, and wrote the manuscript. All authors contributed to the article and approved the submitted version.

## Conflict of Interest

The authors declare that the research was conducted in the absence of any commercial or financial relationships that could be construed as a potential conflict of interest.
