# Supplementary material for: PDLIM7 Synergizes With PDLIM2 and p62/Sqstm1 to Inhibit Inflammatory Signaling by Promoting Degradation of the p65 Subunit of NF-κB
Source: Front Immunol. 2020 Aug 4;11:1559. doi: 10.3389/fimmu.2020.01559 (PMC7417631; doi:10.3389/fimmu.2020.01559)
Supplement: Supplementary file 1 [file Data_Sheet_1.docx]

**Supplementary Figure 1** | Real-time RT-PCR analysis of PDLIM6 expression in mouse tissues and primary immune cells.

**
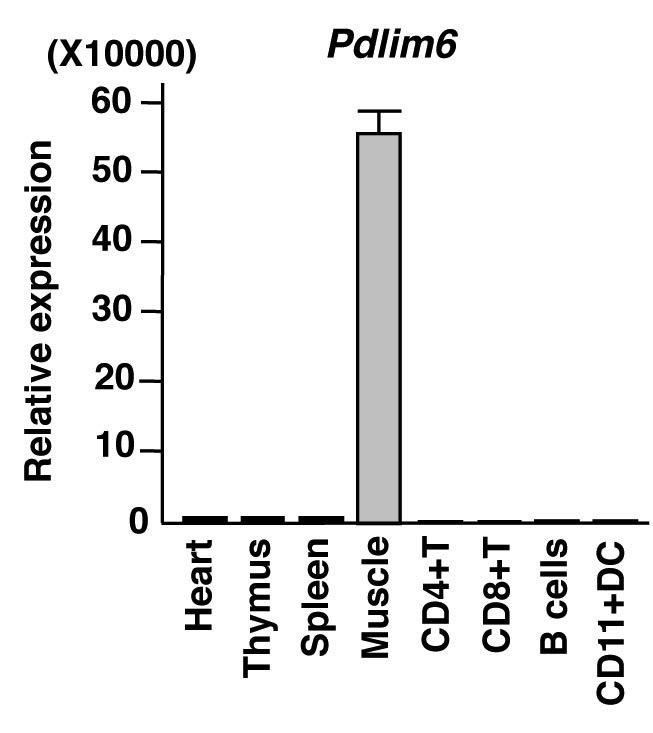
**

The levels of PDLIM6 expression in indicated mouse tissues and primary immune cells were analyzed by real-time RT-PCR analysis. Data are representative of two independent experiments. Shown are the mean values ± SD.

**Supplementary Figure 2** | PDLIM7 promotes proteasomal degradation of nuclear p65 in a LIM3 domain-dependent manner.

**
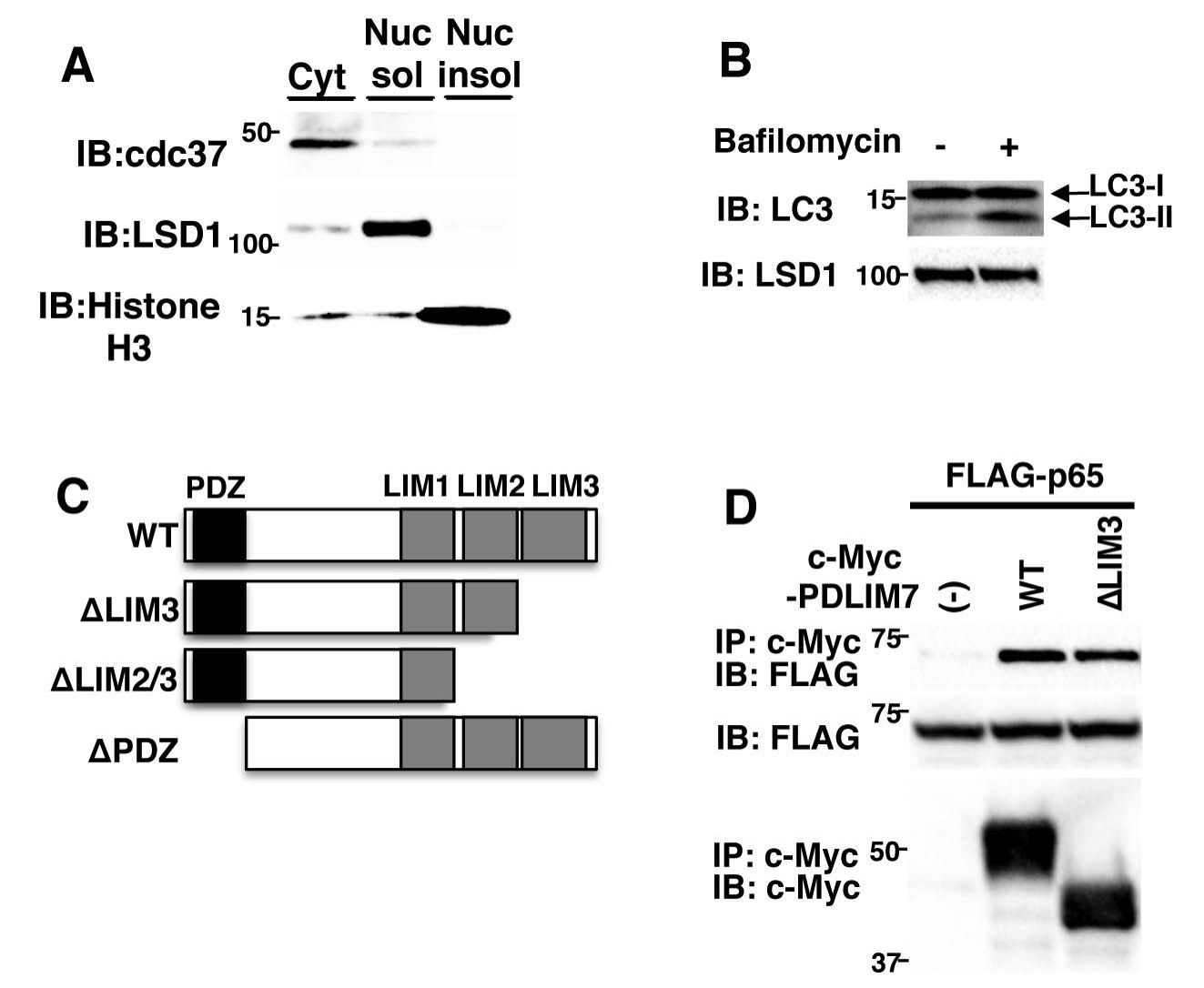
**

(**A**) The purity of the fractions of cytoplasmic and nuclear extracts (soluble and insoluble) in NIH3T3 cells was confirmed by blotting with anti-cdc37 (cytoplasm), anti-LSD1 (nuclear soluble) or anti-Histone H3 (nuclear insoluble) antibody. Western blots are representative of three independent experiments. (**B**) NIH3T3 cells were untreated or treated for 4 h with bafilomycin (100μM) and soluble nuclear extracts were analyzed with anti-LC3 antibody. Western blots are representative of three independent experiments. (**C**) Schematic diagram of the structure of the PDLIM7 mutant lacking the third LIM domain (∆LIM3), second and third LIM domains (∆LIM2/3) and the PDZ domain (∆PDZ). Western blots are representative of three independent experiments. (**D**) 293T cells were transfected with a FLAG-tagged p65 expression plasmid along with or without c-Myc-tagged wild-type or ∆LIM3 PDLIM7. Whole cell extracts were immunoprecipitated with anti-c-Myc and immunoblotted with anti-FLAG antibody. Western blots are representative of five independent experiments.**Supplementary Figure 3** | Purity of the GM-CSF-BMCs fractions

**
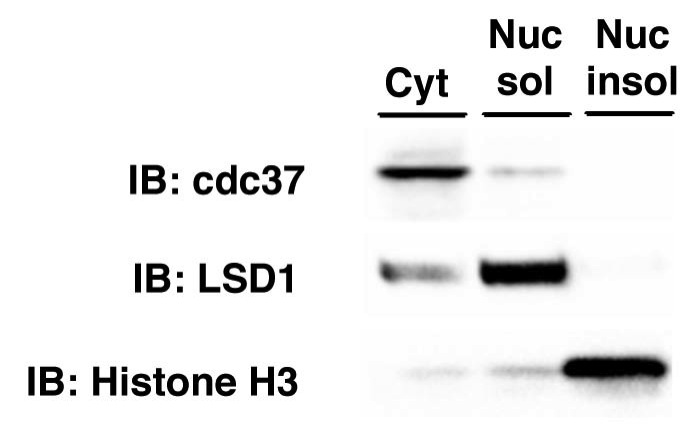
**

The purity of the fractions of cytoplasmic and nuclear extracts (soluble and insoluble) in GM-CSF-BMCs was confirmed by blotting with anti-cdc37 (cytoplasm), anti-LSD1 (nuclear soluble) or anti-Histone H3 (nuclear insoluble) antibodies. Western blots are representative of three independent experiments.

**Supplementary Figure 4** | Reduction of PDLIM7 protein level by siRNA specific for PDLIM7 in 293T cells.

**
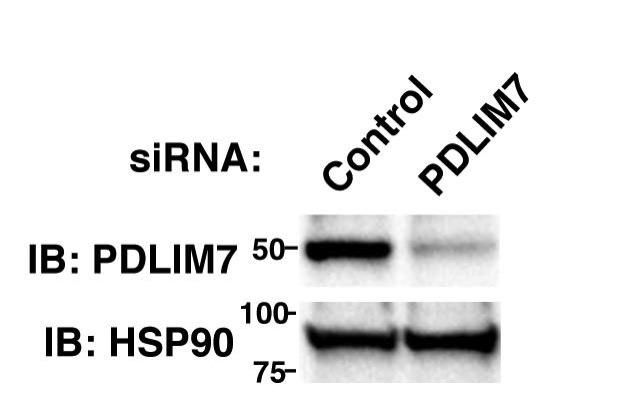
**

293T cells were transfected with control siRNA or PDLIM7-specific siRNA. Two day posttransfection, cells were lysed in RIPA buffer and subjected to immunoblot analysis with indicated antibodies. Western blots are representative of four independent experiments.**Supplementary Figure 5** | PDLIM7-mediated ubiquitination of PDLIM2 does not affect its ability to bind to PDLIM7.

**
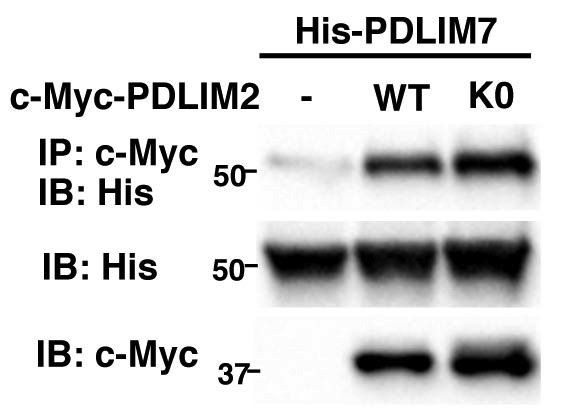
**

293T cells were transfected with His-PDLIM7 along with or without c-Myc-tagged wild-type PDLIM2 or the K0 mutant. Whole cell extracts were immunoprecipitated with anti-c-Myc and immunoblotted with anti-His antibodies. Western blots are representative of three independent experiments. **Supplementary Figure 6** | PDLIM7-mediated ubiquitination of PDLIM2 does not affect its ability to bind to or polyubiquitinate p65.

**
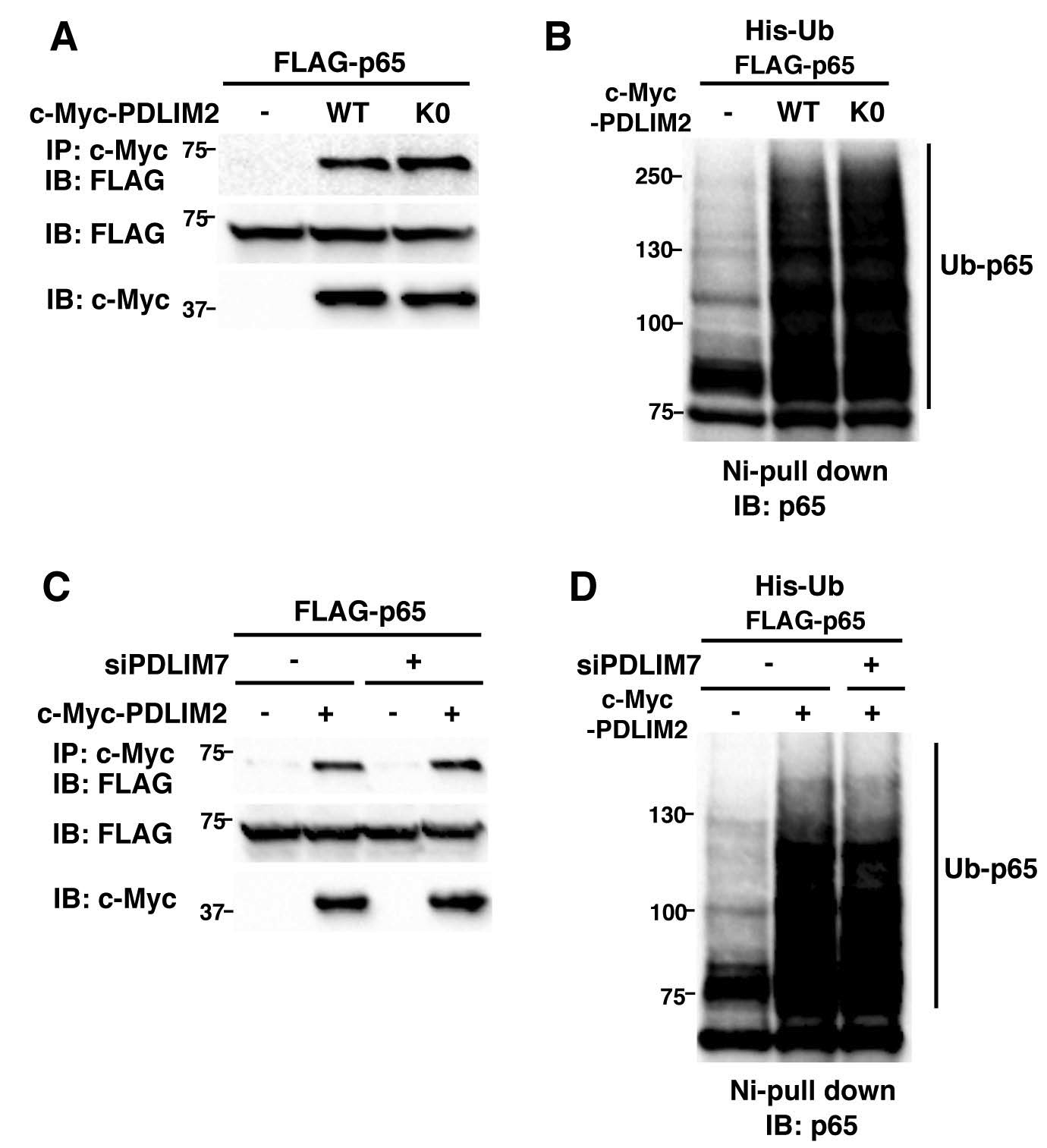
**

**(A)** 293T cells were transfected with FLAG-p65 along with or without c-Myc-tagged WT PDLIM2 or the K0 mutant. Whole cell extracts were immunoprecipitated with anti-c-Myc and immunoblotted with anti-FLAG. Western blots are representative of three independent experiments. **(B)** Ubiquitination assay for p65 in 293T cells cotransfected with plasmids encoding His-Ub and WT PDLIM2 or the K0 mutant, together without or with PDLIM7 and analyzed as in **Fig. 1A**. Western blots are representative of four independent experiments. **(C)** 293T cells were first transfected with control siRNA or PDLIM7-specific siRNA, then transfected with FLAG-p65 along with or without c-Myc-PDLIM2. Whole cell extracts were immunoprecipitated with anti-c-Myc and immunoblotted with anti-FLAG. Western blots are representative of three independent experiments. **(D)** Ubiquitination assay for p65 in 293T cells first transfected with control siRNA or PDLIM7-specific siRNA, then transfected with plasmids encoding His-Ub and PDLIM2, together without or with PDLIM7. Western blots are representative of three independent experiments.

**Supplementary Figure 7** | Reduction of p62/Sqstm1 protein level by siRNA specific for p62/Sqstm1 in BMCs.

**
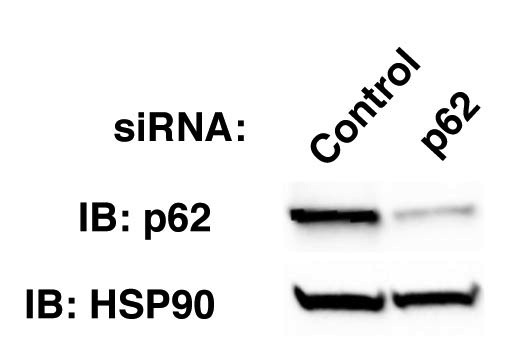
**

Western blot analysis of PDLIM7 expression in whole cell extracts of GM-CSF-BMCs transfected with control or p62/Sqstm1-specific siRNAs. Data are representative of two independent experiments.

**Supplementary Figure 8** | PDLIM7 polyubiquitinate p65 in the absence of PDLIM2 in 293T cells.

**
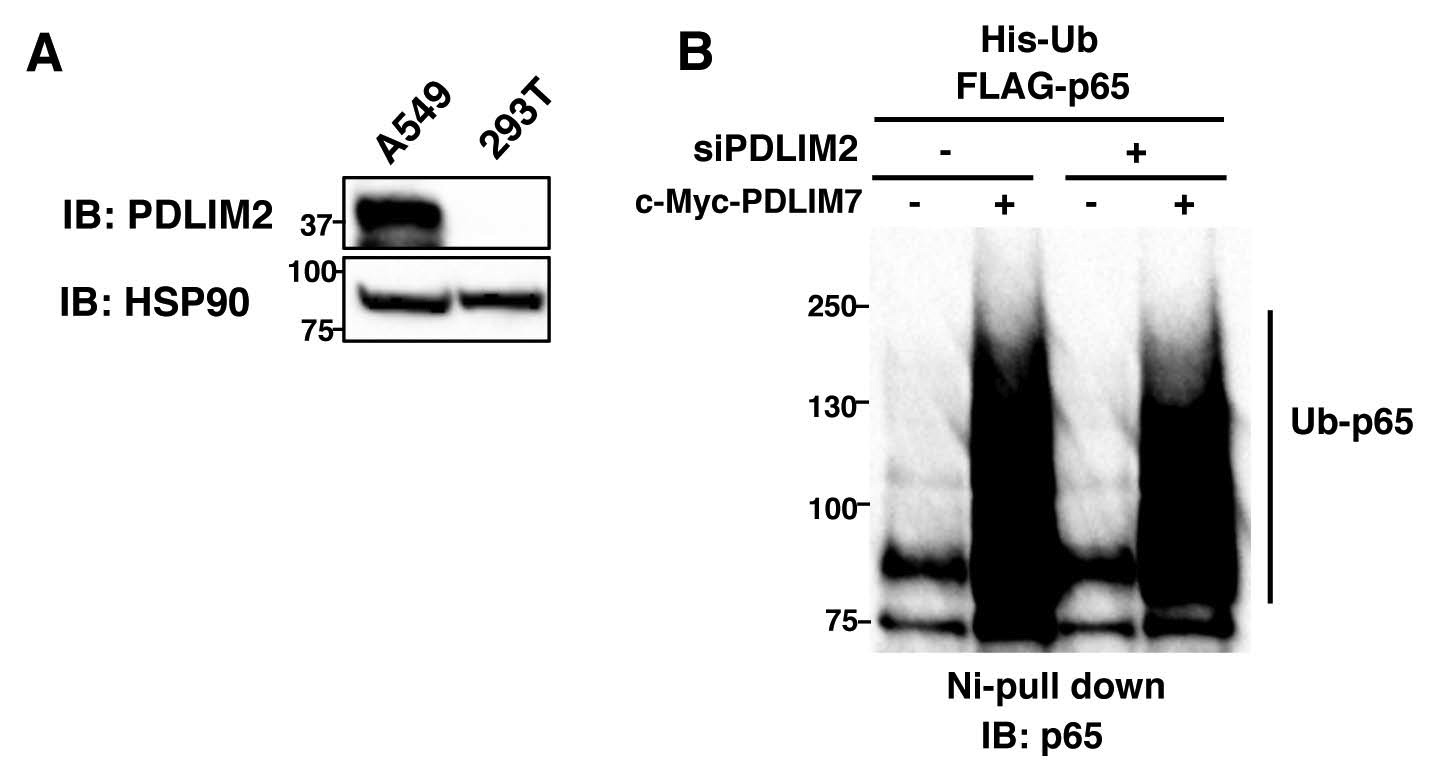
**

**(A)** Western blot analysis of PDLIM2 expression in A549 and 293T cells. Whole cell lysates were subjected to immunoblot with anti-PDLIM2 and HSP90 antibodies. Western blots are representative of two independent experiments. **(B)** Ubiquitination assay for p65 in 293T cells first transfected with control siRNA or PDLIM2-specific siRNA, then transfected with plasmids encoding His-Ub and FLAG-p65, together without or with PDLIM7 and analyzed as in **Fig. 1A**. Western blots are representative of three independent experiments.
